# Readmission and mortality one year after acute hospitalization in older patients with explained and unexplained anemia - a prospective observational cohort study

**DOI:** 10.1186/s12877-016-0284-4

**Published:** 2016-05-24

**Authors:** Jenny Foss Abrahamsen, Anne-Lise Bjorke Monsen, Francesco Landi, Cathrine Haugland, Roy Miodini Nilsen, Anette Hylen Ranhoff

**Affiliations:** Kavli Research Centre for Geriatrics and Dementia, Haraldsplass Deaconess Hospital, Ulriksdal 8, Bergen, 5009 Norway; Department of Nursing Home Medicine, Municipality of Bergen, Norway; Laboratory of Clinical Biochemistry, Haukeland University Hospital, Bergen, Norway; Facoltà di Medicina e chirurgia, Universita Cattoloca del Sacro Cuore, Rome, Italy; Municipality of Bergen, Bergen, Norway; Centre for Clinical Research, Haukeland -University Hospital, Bergen, Norway; Departement of Clinical Science, University of Bergen, Bergen, Norway

**Keywords:** Unexplained anemia, Readmission, Mortality, Elderly, Hospitalization

## Abstract

**Background:**

Few studies have examined whether specific subtypes of anemia in older persons are more related to adverse outcomes such as hospital readmissions and death after acute hospitalization and post-acute care.

**Methods:**

An observational prospective cohort study was conducted between 2011 and 2014. A total of 884 community-dwelling patients, ≥70 years of age were transferred from acute medical and orthopaedic hospital departments to a skilled nursing home where they were examined by comprehensive geriatric assessment and had laboratory tests taken for the investigation of anemia. They were divided into three major groups and compared; 1) no anemia (reference group), 2) explained anemia (renal insufficiency, iron deficiency, vitaminB12/folate deficiency or multifactorial anemia) and 3) unexplained anemia. The groups were compared, and association of anemia with hospital readmission and death was estimated by logistic regression analyses.

**Results:**

Compared to the patients with unexplained anemia (*n*=135), patients with explained anemia (*n*=275) had more often died (22 % vs. 14 %, *p*=0.05) and had more frequenlty been readmitted to hospital (39 % vs. 27 %, *p*=0.03). Compared to the patients without anemia (*n*=474), the patients with explained anemia had increased odds of hospital readmissions (OR = 1.54 (95 % CI: 1.05–2.25), *p*=0.03), while patients with unexplained anemia, (*n*=135), had neither increased odds of hospital readmissions, (OR=0.83, 95 % CI: 0.51–1.34, *p*=0.44) nor death (OR = 0.74, 95 % CI: 0.41–1.31, *p*=0.30), in adjusted regression analysis.

**Conclusion:**

Since no increased risk of hospital readmissions or death was seen in older patients with unexplained anemia in the first year after acute hospitalization, no further invasive investigations might be necessary to investigate the cause of anemia in these patients. A close clinical follow up might be the best way to care for older patients with a mild and unexplained anemia.

## Background

The prevalence of anemia in older persons increases with age, and multifactorial causes related to age-associated renal insufficiency, microscopic bleeding, chronic inflammation, hormonal insufficiency and qualitative bone marrow alterations, like an age-associated diminished hematopoietic stem cell proliferative capacity or myelodysplastic syndrome, play important roles in the pathogenesis [1–3]. Anemia has been associated with several unfavorable outcomes, such as death [4, 5], the worsening of cardiovascular disease [6], cognitive impairment [7], falls [8] and functional dependence [9].

Most studies have focused on the risk of anemia in general, and have not examined whether specific subtypes of anemia are more related to unfavorable outcomes. In approximately one third of older persons no specific cause for the anemia, such as renal insufficiency, nutritional deficiency (iron, vitamin B12, folate) or a combination of these could be found, and the anemia is called unexplained anemia [10–12]. Two studies of community dwelling older people with unexplained anemia have described that people with unexplained anemia did not have any increased risk of mortality as compared to older people without anemia [13, 14]. However, to our knowledge, no study has investigated both the increased risk of hospital readmission and death in hospitalized home-dwelling elderly people with explained and unexplained anemia.

A different clinical outcome in patients with explained and unexplained anemia may imply different threshold for further investigation into the causes of anemia [14], as well as different follow-up and treatment.

## Methods

### Study population

**Fig. 1:**
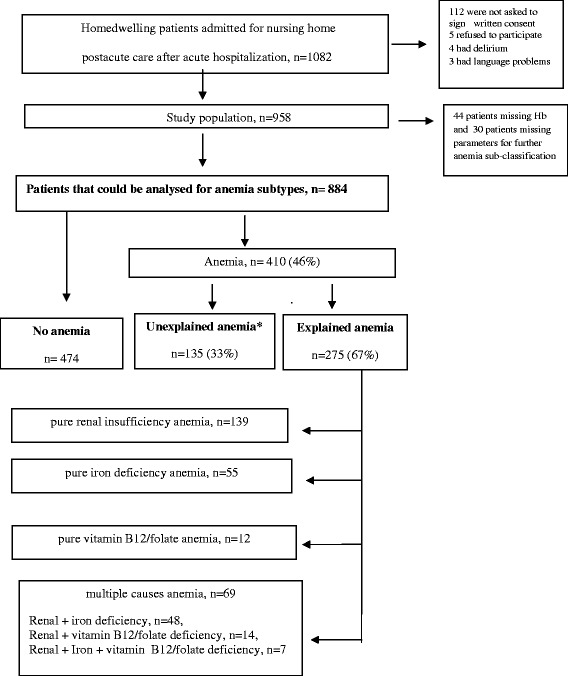
Flow chart describing the patients and subtypes of anemia in the present study

This study is part of a prospective, observational, cohort study, taking place during 2011–2014. It enrolled consecutively older patients who were originally home-dwelling but suffered an acute trauma or illness, were admitted to the hospital and thereafter to a post-acute nursing home ward, before returning to their home [15], In this sub-study 884 patients that could be analysed for anemia and anemia subtypes were included. The patient flow chart is shown in Fig. 1. The patients in the main study, described in detail in a recent paper [15], had been admitted acutely to the two hospitals in Bergen. Both medical patients (from the departments of internal medicine, including cardiology and pulmonology) and orthopaedic patients, with need for further medical treatment or rehabilitation before discharge to own home, were included (Table 1). Most of the orthopaedic patients had suffered a fall, and none were admitted after elective surgery. No patients with active cancer were admitted. The inclusion criteria were as follows: 1) The patients were ≥ 70 years of age and considered to have respiratory and circulatory stability. 2) The hospital doctor expected that the patients would be able to return home within 2 weeks of treatment in the post-acute care unit, and 3) the patients did not have major cognitive impairment or delirium.

**Table 1:** Comparison of baseline characteristics and clinical outcome 12 months after acute hospitalization

	Total sample	No anemia	Anemia
	N=884	474 (54%)	410 (46%)
Demographics			
Age	84 (6.2)	84 (6.2)	85 (6.2)
Male	284	119 (42%)	165 (58%)**
Women	600	355 (59%)	245 (41%)
Receive home care	362 (40%)	196 (42%)	166 (38%)
Admitted after a fall	367 (41%)	137 (38%)	190 (44%)
Admission diagnosis			
Hip fracture	66 (8%)	15 (3%)	51 (12%)**
Other acute trauma	258 (29%)	144 (30%)	114 (28%)
Infection	218 (25%)	116 (25%)	102 (25%)
Heart diseasee	133 (15%)	68 (14%)	65 (16%)
Other medical/pulmonary diseases	209 (23%)	131 (28%)	78 (19%)
Geriatric assessment			
More than 5 diagnosis	528 (63%)	266 (56%)	262 (64%)**
Use > 5 drugs	705 (81%)	361 (76%)	344 (84%)*
Barthel index	75 (60-85)	80 (60-90)	75 (60-85)**
Depression, GDS	7 (4-12)	7 (4-12)	7 (3-12)
Cognition, MMSE	26 (23-28)	26 (23-28)	26 (23-28)
Nutrition, MNA-SF	10 (8-12)	10 (8-12)	10 (8-12)
Laboratory assessments			
Hb, g/dL	12.3 (7.6-19.0)	13.4 (12.0-19.0)	11.0 (7.6-12.9)**
Hb men, g/dL	12.4 (7.6-19.0)	13.8 (13.0-19.0)	11.3 (7.6-12.9)**
Hb women, g/dL	12.3 (8.6-18.0)	13.2 (12.0-18.0	11.0 (8.6-11.9)**
Severe anemi (<10.6g/L)^a^	130 (15%)	0	130 (32%)**
MCVfL	94 (6)	94 (6)	94 (6)
VitB12, pmol/L	506 (331-780)	471 (319 -772)	574 (361-826)**
Folate, nmol/L	16.2 (5.3-45..0)	13.2 (5.0-45.3)	14.8 (5.0-45.3)
Ferritin µg/L	184 (104-350)	179 (101-333)	193 (107-366)
Transferrin receptor, ml/L	3.4 (2.7-4.4)	3.3 (2.7-4.2)	3.4 (2.7-4.6)
CRP, mg/L	18 (1-331)	13 (1-217)	27 (1-331)**
Renal insufficiency^b^	362 (41%)	157 (34%)	205 (50%)**
NT pro-BnP, pmol/L	92 (35-266)	69 (28-188)	134 (45-377)**
1 year follow up			
>2 hospital admissions	229 (33%)	144 (31%)	15 (35%)
Dead	150 (17%)	74 (16%)	77 (19%)

Continuous parameters are presented as mean (standard deviation) and median (interquartile range), categorical data are presented as numbers (percentages)

*GDS* Geriatric Depression scale, range 1-30, *MMSE* Mini Mental Status Examination, *MNA-SF* Mini Nutritional Assessment- Short Form, *CRP* C-reactive protein, *NT pro- BnP* N-Terminal pro brain-type natriuretic peptide, *Hb* Hemoglobin, *MCV* mean corposcular volume.

*p<0.05, **p<0.01 when the group of non-anemic and anemic patients were compared.

^a^Severe anemia defined as Hb concentration in the lower anemia tertile.

^‡^Renal insufficiency was defined as e-GFR (described in methods) < 60 mL/min/1.73 m^2^.

The morning after arrival to the nursing home, they had a venous blood sample drawn. If the results of the blood test indicated that the patients had signs of iron deficiency anemia, and they had no occult blood loss suggestive of gastrointestinal malignancy, they were prescribed iron tablets. If the patients had folate or vitamin B12 deficiency, they were prescribed folate or vitamin B12 substitution. At discharge, all patients with anemia were asked to follow up with their general practitioner.

### Data collection

The data on patient’s demographic and baseline clinical characteristics were obtained from hospital records. During the first week in the nursing home comprehensive geriatric assessment was performed on >90 % of the patients using the Barthel index sumscore (BI) [16], the Norwegian version of the Mini Mental Status Examination, MMSE [17, 18], Geriatric Depression Scale GDS [19], and Mini Nutritional Assessment- Short Form; MNA-SF [20].

Data regarding hospital readmissions were obtained from electronic patient registers and digital health records at Haukeland and Haraldsplass hospitals (data obtained from 80 % of the patients). Mortality information (whether the patients were alive or dead 12 months after hospital discharge), was collected from the patient administrative system in the municipality (data obtained from 98 % of the patients).

### Laboratory measurements

Blood was collected into EDTA Vacutainer Tubes (Becton Dickinson) and hematological parameters (Hemoglobin level (Hb), MCV (mean corpuscular volume), white blood cells (WBS) and platelets,) were analyzed with an automated hematology analyzer (ADVIA 120, Bayer Diagnostics, Tarrytown, NY, USA).

Serum was obtained by collecting blood into Vacutainer Tubes with no additive (Becton Dickinson). Serum levels of creatinine (CV 1.8 %), C-reactive protein (CRP) (CV 3.3 %) and soluble transferrin receptor (TfR) (CV 3.5 %) were analyzed with Modular P, levels of cobalamin (vitamin B12) (CV 4.4 %), folate (CV 7.5 %) and NT-proBNP (N-Terminal pro brain-type natriuretic peptide) (CV 4.8 %) with Modular E and serum ferritin (CV 4.5 %) by Modular PP (F. Hoffman-La Roche Ltd, Basel, Switzerland).

The Modification of Diet in Renal Disease (MDRD) Study equation; GFR (glomerular filtration rate) (mL/min/1.73 m^2^) = 175 × (S_cr_)^-1.154^ × (Age)^-0.203^ × (0.742 if female), was used for estimating GFR. The equation does not require weight or height variables because the results are reported normalized to 1.73 m^2^ body surface area, which is an accepted average adult surface area. The equation has been validated in Caucasian populations between the ages of 18 and 70 with impaired kidney function (eGFR < 60 mL/min/1.73 m^2^) and has shown good performance for patients with all common causes of kidney disease [21].

Iron deficiency was assessed by measuring Ferritin and TfR, since other studies have demonstrated that these measurements give a more sensitive measure of iron-deficiency than the more commonly used Transferrin saturation (Serum-iron/Total Iron Binding Capacity (TIBC)) [22, 23]. Transferrin receptor rise, independent of inflammation, when the iron level available for erythropoiesis decrease [23].

N-Terminal pro brain-type natriuretic hormone (pro-BnP) (level > 225 pg/L [24]) has been demonstrated to be a sensitive marker clinical cardiovascular disease [24] and renal dysfunction [25], and was included as a measure of these comorbidities.

### Definitions and subdivision of anemia subtypes

- Anemia was classified according to the World Health Organization (WHO) definition as a haemoglobin concentration of less than 12 g/dL in women and less than 13 g/dL in men.
- Severe anemi was defined as having a haemoglobin concentration in the lowest gender-specific tertile of the anemia patients. The 33.3 percentile of haemoglobin concentration both for men and women with anemia was 10.6 g/dL, and this value was used as cut-off for defining patients with severe anemia.
- Renal anemia was defined as anemia combined with eGFR < 60 ml/min/1.73 m^2^ and normal levels of ferritin, folate and cobalamin
- Iron deficiency anemia was defined as anemia combined with ferritin < 35 μg/L or TfR > 4.5 mg/L
- Vitamin B12 or folate anemia was defined as anemia with vit B12 < 200 pmol/L or folate < 8 nmol/L, and normal levels for creatinine and ferritin and TfR
- Multifactorial anemia was defined as anemia with more than one of the above explainable causes of the anemia.
- Explainable anemia were defined as either renal anemia, iron deficiency anemia, vit B12/folate deficiency anemia or multiple cause anemia
- Unexplainable anemia was defined as anemia without any known cause listed for the explained anemias.

### Statistical analyses

Continuous data with a normal distribution was presented as mean (standard deviation) and compared with two sample *t*-test. Continuous data with a non-normal distribution was presented as median (interquartile range) and compared with the Mann–Whitney *U* test. Categorical data was presented as numbers (percentages) and compared with the chi-square test.

For identifying the clinical characteristics that were independently associated with hospital readmissions and death, odds ratios (ORs) with 95 % confidence intervals (CIs) were estimated using logistic regression models. The characteristics associated with *p* < 0.25 in univariate analysis were noted as likely predictors and included in multivariate, adjusted logistic regression models. When analysing hospital readmission as the outcome, the covariates age, >5 diagnoses, Barthel index, and the influence of five different admission diagnoses groups were included in the multivariate analysis. When analyzing mortality as outcome, the additional covariates male sex, hemoglobin concentration, renal failure and MMSE score were also included in multivariate analysis.

All analyses were performed using the Statistical Package for Social Science (IBM SPSS), version 20 for Windows.

## Results

### Baseline patient characteristics and difference between patients with and without anemia

As shown in Table 1, the patients in general had rather good physical, cognitive and nutritional status, but still wide inter-individual variations were present in geriatric assessment tests.

Anemia based on the WHO criterias was present in 410 (46 %) of the patients while severe anemia was present in 130 (15 %) of the patients.

Compared to the patients without anemia, the anemic patients were older, more often male sex, more of them had suffered a hip fracture, they had worse ADL function (lower BI), they had more diagnoses and were using more medications. Their CRP and pro-BnP levels were higher, and more of them had renal insufficiency.

There was no significant difference in the prevalence of hospital readmission or death in patients with and without anemia (Table 1). As shown in Table 2, no association was shown between anemia and death and anemia and hospital readmissions in regression analysis. However, sub-analyses of patients with severe anemia (Hb < 10.6), demonstrated an increased odds for death but no increased odds of hospital readmission, when patients with severe anemia were compared to non-anemic patients.

**Table 2:** Unadjusted and adjusted Odds Ratios for hospital readmission and death 12 months after acute hospitalization

	Hospital readmission^a^							Death^b^				
	Univariate			Multivariate			Univariate			Multivariate		
	OR	95 % CI	*p*	OR	95 % CI	*p*	OR	95 % CI	*p*	OR	95 % CI	*p*
Anemia^c^	1.20	0.87–1.65	0.26	1.42	0.96–2.09	0.08	1.24	0.88–1.77	0.22	1.24	0.83–1.84	0.30
Severe anemia,^d^	0.92	0.59–1.44	0.73	1.13	0.66–1.94	0.66	1.88	1.21–2.93	0.005	1.89	1.11–3.22	0.02
Unexplained anemia^e^	0.83	0.51–1.34	0.44	0.95	0.55–1.64	0.83	0.74	0.41–1.31	0.30	1.04	0.54–2.02	0.90
Explained anemia^f^	1.42	1.00–2.01	0.05	1.54	1.05–2.25	0.03	1.53	1.04–2.23	0.03	1.07	0.63–1.83	0.79

*OR* odds ratio, *CI* confidence interval, *Hb* hemoglobin concentration

^a^Adjustment for age, > 5 diagnoses, Barthel index, influence of 5 different admission diagnoses

^b^Adjustment for age, > 5 diagnoses, Barthel index, influence of 5 different admission diagnoses, gender, MMSE, renal insufficiency

^c^Comparing patients with or without anemia, defined by WHO

^d^Comparing patients with and without severe anemia, defined as Hb in lowest gender –specific anemia tertile, (<10.6 g/dL)

^e^Comparing patients with unexplained anemia versus patients without anemia (WHO defined)

^f^Comparing patients with explained anemia versus patients without anemia (WHO defined)

### Difference in baseline characteristics and outcome in patients with subgroups of explainable anemia

As indicated in Fig. 1, one or more explainable cause (s) could be found for 275 (67 %) of all the patients with anemia. Renal insufficiency was the most common cause, and was present in 208 (76 %) of the patients with explained anemia (139 pure renal anemias + 69 multifactorial anemias), more often in men than in women.

As shown in Table 3, the patients with pure iron deficiency anemia had the lowest hemoglobin and 50 % of them had been admitted to hospital the first year. Only 9 % of them had been operated on for a hip fracture.

**Table 3:** Baseline characteristics and outcome 12 months after acute hospitalization in patients with different anemia subtypes

	No anemia	Explained anemia subtypes, *n*=275			
		Renal anemia^a^	Iron deficieny^b^	B12/folate deficiency^c^	Multifactorial^d^
	*N*=474	*N*=139	*N*=55	*N*=12	*N*=69
Age	84 (6.0)	86 (6.3)	85 (6.0)	83 (7.5)	84 (6.0)
Male sex	119 (25 %)	52 (37 %)**	19 (35 %)	8 (67 %)**	35 (51 %)**
Barthel index	80 (60–90)	75 (60–85)	65 (55–83)**	75 (70–80)	80 (55–90)
Diagnosis					
hip fracture	15 (3 %)	10 (7 %)	5 (9 %)	3 (25 %)	3 (4 %)
other orthopaedic trauma	144 (30 %)	39 (28 %)	20 (36 %)	2 (17 %)	10 (15 %)
acute infection	116 (25 %)	37 (27 %)	12 (22 %)	2 (17 %)	20 (29 %)
heart disease	68 (14 %)	24 (18 %)	8 (16 %)	3 (20 %)	15 (22 %)
other medical diseases	131 (28 %)	29 (21 %)	10 (19 %)	3 (20 %)	21 (30 %)
Laboratory parameters					
Hb, g/dL	13.6 (1.1)	11.0 (0.9)**	10.8 (0.8)**	11.5 (0.9)**	11.0 (1.1)**
MCV, fL	94 (5.2)	94 (5.3)	91 (7.0)**	94 (7.5)	95 (5.2)*
Vitamin B12, pmol/L	471 (331–780)	601 (403–841)**	581 (393–765)	282 (177–1009)	541 (328–775)
Folat, nmol/L	16 (11–24)	17 (12–27)	19 (13–29)	7 (6–11)**	13 (8–19)
Ferritin, μg/L	179 (101–333)	228 (119–400)*	120 (45–201)**	252 (175–424)	168 (74–294)
Transferrin receptor, ml/L	3.3 (2.7–4.2)	3.1 (2.5–3.6)*	5.7 (4.8–6.9)**	3.2 (3.0–3.7)	5.2 (4.5–6.9)**
CRP, mg/L	13 (5–30)	22 (9–48)**	26 (14–57)**	31 (22–78)**	27 (12–64)**
NTpro-BnP	70 (28–188)	294 (104–636)**	110 (50–263)*	86 (33–200)	290 (55–963)**
12 months follow up					
> 2 hospital admissions	124 (26 %)	36 (39 %)	21 (50 %)**	2 (18 %)	23 (41 %)*
Dead	74 (16 %)	25 (21 %)	12 (23 %)	2 (18 %)	16 (23 %)

^a^anemia + estimated GFR (described in methods) < 60 mL/min/1.73 m^2^

^b^anemia + ferritin < 35 ug/L or transferrin receptor > 4.5 mg/L

^c^anemia + Vit B12 < 200 pmol/L or folate <8 nmol/L

^d^renal anemia and iron deficiency anemia (*n*=40), renal anemia + B12/folater deficiency anemia (*n*=11) or renal anemia + iron deficiency anemia + B12/folate deficiency anemia (*n*=6)

Continous parameters are presented as mean (standard deviation) or median (interquartile range), categorical data are presented as numbers (percentages)

*Hb* haemoglobin, *MCV* mean corpuscular volume, *CRP* C-reactive protein, *NT pro- BnP* N-Terminal pro brain-type natriuretic peptide, *p < 0.05, **p < 0.01 when patients with each of the anemia subtypes were compared to patients without anemia

### Difference in baseline characteristics and outcome in patients with unexplained and explained anemia

As shown in Table 4, the hemoglobin was comparable between the patients with unexplainable and explainable anemias, and both groups had significantly increased CRP, as compared to patients without anemia.

**Table 4:** Characteristics and outcome 12 months after acute hospitalization in patients with explained and unexplained anemia

	No anemia	Unexplained anemia	Explained anemia	Unexplained vs explained anemia
	*N*=474	*N*=154	*N*=256	*p*-value
Demographics				
Age	84 (6.0)	84 (6.2)	85 (6.2)	0.08
Male sex	119 (25 %)	51 (38 %)**	114 (42 %)**	0.48
Receive home care	196 (41 %)	43 (32 %)	114 (42 %)	0.07
Admitted after a fall	177 (38 %)	73 (55 %)**	103 (38 %)	0.002
Diagnosis				
Hip fracture	15 (3 %)	30 (12 %)**	21 (8 %)*	0.001
Other acute trauma	144 (30 %)	51 (33 %)	63 (25 %)	0.06
infection	116 (24 %)	36 (23 %)	66 (26 %)	0.63
heart disease	68 (14 %)	16 (10 %)	49 (19 %)	0.02
other medical	131 (28 %)	21 (14 %)**	57 (22 %)	0.03
Geriatric assessment				
More than 5 diagnosis	266 (56 %)	76 (60 %)	186 (71 %)**	0.02
Use > 5 drugs	361 (77 %)	100 (75 %)	244 (90 %)**	<0.001
Barthel index	80 (60–90)	70 (60–85)**	75 (60–85)*	0.37
MMSE	26 (23–28)	26 (23–28)	26 (23–28)	0.78
GDS	7 (4–12)	7 (3–11)	6 (4–11)	0.88
MNA-SF	10 (8–12)	10 (7–11)	10 (8–11)	0.42
Laboratory investigations				
Hb g/dl	13.6 (1.1)	11.0 (1.0)**	11.0 (0.9)**	0.22
Severe anemia (Hb < 10.6 g/L)^a^	0	38 (28 %)**	92 (36 %)**	0.29
MCV fL	94 (5.5)	95 (5.2)	93 (6.1)	0.001
Ferritin	179 (101–333)	204 (138–399)**	171 (93–336)	0.007
Transferrin Receptor (TfR)	3.3 (2.7–4.2)	3.0 (2.4–3.4)**	4.1 (3.0–5.5)**	<0.001
CRP mg/L	13 (5–30)	31 (10–63)**	27 (12–53)**	0.60
Pro-BnP > 225 pmol/L	90 (20 %)	21 (16 %)	110 (42 %)**	<0.001
Renal insufficiency^b^	157 (34 %)	0**	205 (75 %)**	<0.001
Iron deficiency^c^	91 (21 %)	0**	110 (43 %)**	<0.001
12 months follow up				
>2 hospital admissions	114 (31 %)	29 (27 %)	86 (39 %)*	0.03
Dead	74 (16 %)	21 (14 %)	55 (22 %)*	0.05

*GDS* Geriatric Depression scale 1–30, *MMS* Mini Mental Status Examination, *MNA-SF* Mini Nutritional Assessment- Short Form, *CRP* C-reactive protein, *NT pro- BnP* N-Terminal pro brain-type natriuretic peptide

^a^Severe anemia defined as Hb concentration in the lower anemia tertile

^b^Renal insufficiency was defined as e-GFR (described in methods) < 60 mL/min/1.73 m^2^

^c^Iron deficiency defined as anemi + ferritin < 35ug/l or TfR > 4.5 mg/L

Continous parameters are presented as mean (standard deviation), and median (interquartile range), Categorical data are presented as numbers (percentages)

*p < 0.05, **p < 0.01 when the group of patients with unexplained and explained anemias each were compared with patients without anemia

Compared to the patients with explained anemia, the patients with unexplained anemias were more often women, were admitted after a fall and had suffered a hip fracture, but they had fewer diagnoses and were using less drugs. None of them (per definition) had iron deficiency nor renal insufficiency, and most of them did not have elevated NT-pro BnP, indicating no heart failure. The patients with unexplained anemia had no more hospitals admission or death than patients with no anemia. As shown in Table 2, no significant association was demonstrated between unexplained anemia and hospital admissions and unexplained anemia and death. When the analysis was repeated for patients with severe unexplained anemia, the results were not materially different.

As shown in Table 4, patients with explained anemia had more frequently been readmitted to hospital or had died during the first 12 months than patients without anemia. Furthermore, a significant association was demonstrated between explained anemia and hospital readmission in adjusted regression analysis, while no significant association between explained anemia and death was demonstrated in adjusted regression analysis (Table 2).

## Discussion

The main finding in the present study is that no increased risk of 1-year mortality and hospital readmissions could be demonstrated in older patients that had unexplained anemia, when they were discharged from acute hospitalization, compared to patients with no anemia. On the other hand, more patients with explained anemia died during the first year, and they had a higher risk of hospital readmissions, compared to patients without anemia. These findings imply that it is clinically important to distinguish between these two groups of anemias, because patients with unexplained and explained anemia may require different follow up and treatment.

To our knowledge no earlier studies have reported risk of hospital readmissions and death in older patients with explained and unexplained anemia, one year after acute hospitalization. However, our results are in agreement with the Leiden 85 plus study, which demonstrated no increased risk of mortality in 86 year old community dwelling persons with unexplained anemia compared to no anemia, after a 6-years follow-up [14]. Similarly, the Women’s Health and Aging Study I, which included moderately to severely disabled women > 65 years of age, could not demonstrate increased mortality after five years in patients with unexplained anemia compared to non-anemic women [13].

Several other studies characterize a group of explained anemias related to chronic disease/chronic inflammation [10, 13, 26]. However, the definition for this anemia subtype is not uniform [11, 27]. Since a majority of our patients had chronic diseases, all of them had suffered an acute trauma or illness, and 80 % of them had an elevated CRP > 5, we decided, in line with the Leiden study [14], not to conduct any subgrouping of anemia patients with acute/chronic inflammation or chronic disease, but rather subdivide patients with explained and unexplained anemias based on simple, standardized laboratory measurements. The fractions of patients with unexplained and explained anemia in our study are in accordance with other studies of older patients living in the community [13, 14, 27], and with a small cohort of older patients from an acute medical hospital unit [28], and an university anemia clinic [11].

Our patients, with a mean age of 85 years, were living in the community, and 80 % of them returned home after a short stay in the hospital followed by post-acute care [15]. Thus they may be partly comparable to both anemic persons living in the community [3, 13, 14, 29] and anemia patients from hospital cohorts [28, 30–32], but not strictly representative of a normal, home-dwelling elderly population. The overall prevalence of anemia in our study (47 %) was similar to other studies reporting anemia in a hospital setting [28, 32, 33], but lower than reported from long term care [5].

While no overall risk of mortality was demonstrated in the anemic population when applying the WHO definition, a significant association with mortality was demonstrated for the anemia patients with a hemoglobin concentration in the lower anemia tertile (10.6 g/dL). This is partly in agreement with a large study of 17 030 community dwelling older patients, reporting increased mortality in patients with a hemoglobin < 11gd/L [4], and with a previous study from long-term care demonstrating an association between high levels of hemoglobin and better survival [5]. Unfortunately, no recording of the causes of death was recorded, thus we were unable to investigate whether different types of anemi were related to different causes of death.

Like other studies on anemia, renal insufficiency and iron deficiency were the most common causes of the explainable anemias, often together [14, 27]. In our study patients with iron deficiency anemia had the lowest Hb and the most frequent hospital readmissions, compared with the other anemia subgroups. Thus, the recognition of patients with iron deficiency anemia is particularly important to diagnose, as these patients may be more prone to hospital readmissions, and this type of anemia may be susceptible to treatment.

Clinical baseline differences were demonstrated between patients with unexplained and explained anemias. While both patient groups had rather mild anemia and elevated CRP, the patients with unexplained anemias more often had suffered a fall, even though they had fewer diagnoses, were using fewer drugs and had a lower pro-BnP, indicative of less cardiovascular disease. This suggests that the unexplained anemia in these patients might be more associated with frailty and the pro-inflammatory state of ageing, whereas the explained anemia is more related to multimorbidity. Although the reason for the observed differences in hospital readmission and mortality between patients with explained and unexplained anemia is not obvious from our study, we may speculate that the influence of both renal dysfunction, and possible heart failure, in combination with anemia, may influence the increased readmssion and mortality seen in the patients with explained anemia.

The findings in the present study may have clinical importance for the follow up and treatment of older patients diagnosed with anemia. When anemia is found in the elderly, it is important to determine if it is explained by age related physiological degeneration of bone marrow function or if there is an underlying disease of which the treatment can improve the anemia. We recommend that all patients with a subnormal hemoglobin level should have a repeated simple biochemical standard blood test including the measurements of Hb, MCV, eGFR, Ferritin, s-TfR (or se-Fe and TIBC), CRP, B12 and folate, to indicate whether the patients have an explained or unexplained anemia. If the patients have an explained anemia, further care should be directed towards handling these explained causes. For patients with iron deficiency, further search for GI disease or cancer should be sought before iron administration. If the patients have a renal anemia, particular care should be directed towards the use of nephrotoxic drugs and drug dosages, and treatment with Erythropoietin might be considered if blood serum levels are not elevated. For older patients with unexplained anemia, we support the conclusion from the Leiden study, that no further invasive investigations might be necessary in determining the cause of the anemia [14]. However, we recommend that these patients are followed clinically, and that a screening test for frailty is included, as the anemia may precede, predispose or accelerate the development of frailty [34–36].

A limitation of the present study is that the follow up time of 12 months might be too short to assess the mortality risk in anemia patients. Furthermore, no formal assessment of multi-morbidity or frailty was done. The strength of our study is the prospective follow-up setting and the inclusion of a complete baseline geriatric evaluation, laboratory investigations and a one year follow-up. This enables new knowledge of both the clinical patient characteristics and the future clinical unfavourable outcomes, in older patients with unexplained and explained anemia.

## Conclusion

In contrast to patients with an explained anemia, older anemic patients ≥ 70 years of age, without the presence of renal insufficiency, iron deficiency and B12/folate deficiency, had no increased risk of hospital readmission and death the first year after an acute hospitalization, compared to patients without anemia. This suggests that no further invasive investigations might be necessary to investigate the cause of the anemia, and that a close clinical follow up might be the best way to care for these older patients with a mild and unexplained anemia.

## Abbreviations

BI, Barthel index sumscore; CI, confidence interval; CRP, C-reactive protein; GDS, Geriatric Depression Scale; GFR, glomerular filtration rate; Hb, hemoglobin level; MCV, mean corposcular volume; MMSE, Mini Mental Status Examination; MNA-SF, mini nutritional assessment-short form; NT-pro BNP, N-Terminal pro brain-type natriuretic peptide; OR, odds ratio; TfR, transferrin receptor; WBS, white blood cells.
